# Signaling Potential Therapeutic Herbal Medicine Prescription for Treating COVID-19 by Collaborative Filtering

**DOI:** 10.3389/fphar.2021.759479

**Published:** 2021-12-24

**Authors:** Fan Yang, Qi Zhang, Zhongshang Yuan, Saisai Teng, Lizhen Cui, Fuzhong Xue, Leyi Wei

**Affiliations:** ^1^ Department of Epidemiology and Biostatistics, School of Public Health, Cheeloo College of Medicine, Shandong University, Jinan, China; ^2^ Institute for Medical Dataology, Cheeloo College of Medicine, Shandong University, Jinan, China; ^3^ School of Software, Shandong University, Jinan, China; ^4^ Joint SDU-NTU Centre for Artificial Intelligence Research (C-FAIR), Shandong University, Jinan, China

**Keywords:** collaborative filtering, COVID-19, SARS-CoV-2 proteins, traditional herbal medicine, small molecule docking

## Abstract

Severe acute respiratory syndrome coronavirus 2 (SARS-CoV-2) has aggressed in more than 200 countries and territories since Dec 2019, and 30 million cases of coronavirus disease 2019 (COVID-19) caused by SARS-CoV-2 have been reported, including 950,000 deaths. Supportive treatment remains the mainstay of therapy for COVID-19. There are no small-molecule–specific antiviral drugs available to prevent and treat COVID-19 until recently. Herbal medicine can facilitate syndrome differentiation and treatment according to the clinical manifestations of patients and has demonstrated effectiveness in epidemic prevention and control. The National Health Commission (NHC) of China has recommended “three TCM prescriptions and three medicines,” as a group of six effective herbal formulas against COVID-19 in the released official file “Diagnosis and Treatment Protocol for COVID-19 Patients: Herbal Medicine for the Priority Treatment of COVID-19.” This study aimed to develop a collaborative filtering approach to signaling drug combinations that are similar to the six herbal formulas as potential therapeutic treatments for treating COVID-19. The results have been evaluated by herbal medicine experts’ domain knowledge.

## Introduction

The novel coronavirus disease 2019 (COVID-19) pandemic caused by SARS-CoV-2 continues to cause a rush threat to global health. The outbreak that started in early December 2019 has spread worldwide. As of October 2nd, 2021, the overall number of patients confirmed to have the disease has exceeded 235,063,766 in more than 180 countries, though the number of people infected is probably much higher. More than 4,805,700 people have died from COVID-19.^1^ This pandemic is ongoing, so quickly identifying new preventive and therapeutic agents is a top priority.

Although SARS-CoV-2 is widespread and causes multiple organ damage, no specific antiviral drugs or vaccines are currently available. Development of these treatments usually takes a long period, meaning that a more immediate treatment should be found if at all possible. A report issued by the World Health Organization (WHO) presented that the herbal medicine could be a potentially valuable resource to this end.^2^ The effectiveness of herbal treatment in controlling contagious disease is demonstrated during the 2003 severe acute respiratory syndrome (SARS) outbreak (Chen and Nakamura, 2010).

The summarized clinical features of COVID-19 are shown in Table 1. The SARS-CoV-2 infected patients are categorized into different types such as asymptomatic cases, suspected cases, and confirmed cases (Li et al., 2020a). In order to analyze infected patients directly, the clinical features of COVID-19 are only taken from confirmed symptomatic infected patients without considering the asymptomatic and suspected patients. The confirmed cases can be divided into mild cases, common cases, and severe cases because of their different clinical manifestations. Patients with mild symptoms are characterized by low-grade fever and mild fatigue. The most common features of COVID-19 are fever, fatigue, cough, and bilateral distribution of ground-glass shadows under chest CT scan imaging (Ning et al., 2020). In addition, some patients also exhibited runny nose, sore throat, and diarrhea (Huang et al., 2020). Symptoms of dyspnea and hypoxemia appeared in severe cases after a week onset, rapidly deteriorating into ARDS, septic shock, coagulation dysfunction, and multiple organ failure (Huang et al., 2020).

**TABLE 1:** Clinical features of COVID-19.

	Asymptomatic cases	Suspected cases	Confirmed cases		
			Mild cases	Common cases	Severe and critical cases
Fever	−	+	+	+	+/#
Fatigue	−	+	+	+	+
Non-productive cough	−	+	+	+	+
Diarrhea	−	+	+	+	+
Dyspnea	−	−	-	−	+
White blood cell counts	−	−/ ↓	−/ ↓	−/ ↓	↓
Lymphocyte counts	−	−/ ↓	−/ ↓	−/ ↓	↓
Hyoxemia	−	−	−	−	+
ARDS	−	−	−	−	+
Septic shock	−	−	−	−	+
Coagulation disorders	−	−	−	−	+
Multiple organ dysfunction	−	−	−	−	+
Pneumonia	−	+	+	+	+
Pulmonary imaging	−	+	+	+	+
SARS-CoV-2 nucleic acid	+	−	+	+	+
Specific IgM antibody	+	−	+	+	+
Specific IgG antibody	+	−	+	+	+

Note: “+” means positive index; “-” means negative index; “#” means some severe patients with no fever symptom; “ ↓ ” means decreasing index.

The aforementioned symptoms of COVID-19 are similar to those explained in the medical book of *HuangDi Neijing* (Cavalieri and Rotoli, 1997), for plague category, being highly infectious and epidemic. COVID-19 could be classified into cold-dampness epidemic (a disease caused by internal abundance of the cold-damp pestilential pathogen) (Zhao et al., 2021). It is proposed that the direct cause of this disease is the invasion of evil *Qi*, and basic cause is the insufficiency of vital *Qi*, as well as the abnormal external environment at the end of 2019 (Zeng, 2020). Referring from the epidemic, COVID-19 can be divided into four stages: the early stage with symptoms of cold-dampness evils attacking the lung and spleen, the middle stage with symptoms of cold-dampness evils obstructing the lung and spleen, the late stage with symptoms of cold-dampness evils closing the lung and injuring spleen, and the recovery stage with symptoms of Qi-deficiency of the lung and spleen (Zeng, 2020).

**FIGURE 1:**
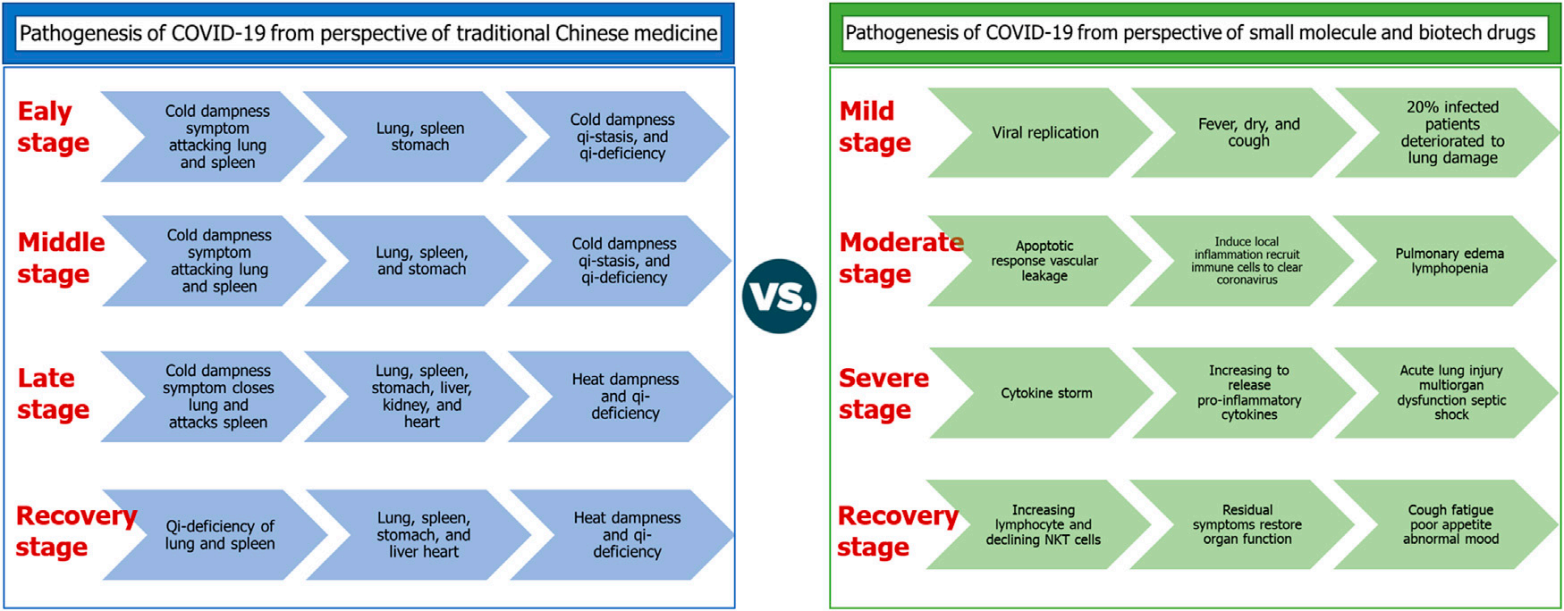
Compared pathogenesis of COVID-19 from herbal medicines and small-molecule drugs.

Ren et al. (2021) compared the corresponding pathogenesis of SARS-CoV-2 infection with the perspective of small-molecule drugs, which is also shown in Figure 1. In the mild stage, SARS-CoV-2 replication occurred in the trachea, which may be incubated for 5–6 days (Wölfel et al., 2020). After that, there is a mild symptom for 80% of infected patients, mainly including fever and dry cough, which disappeared spontaneously within 6–10 days (Li et al., 2020b; Chan et al., 2020), whereas around 20% of patients developed viral infection from the trachea to the lungs (Li et al., 2020b). SARS-CoV-2 binds with targets in alveolar epithelial cells such as ACE2 and TMPRSS2, and induces apoptosis response associated with vascular leakage (Dong et al., 2020). This leakage causes the first wave of local inflammation and recruits immune cells from the blood into the lungs, thereby eliminating extracellular viruses and destroying infected cells (Shi et al., 2020). In this stage, the disease may rapidly develop into severe illness manifested as acute respiratory distress syndrome, acute lung injury, multiple organ dysfunction, and septic shock (Perico et al., 2021). During the recovery stage, it is reported that some patients still have clinical manifestations such as cough, fatigue, poor appetite, and abnormal mood, which need more time to be recovered completely. In terms of the comprehensive contrastive analysis, herbal medicines have a similar treating process with small-molecule drugs.

A definitive therapeutic agent for managing COVID-19 has not been recommended for humans until now. Current preventive and treatment efforts for COVID-19 have focused on developing vaccines and specific therapeutic agents targeting SARS-CoV-2 (Dhama et al., 2020). Although Food and Drug Administration (FDA)–approved antiviral drugs and several types of vaccines are currently available, many alternative treatments are still being proposed as an adjunctive treatment for COVID-19. It has been reported that herbal medicines could be considered an alternative approach for the treatment and prevention of COVID-19 (Remali and Aizat, 2020). More systematic review articles also concluded that herbal medicine showed significant effects in increasing the total effective rate and alleviating the symptoms (Ang et al., 2020; Liu et al., 2020).

Although the use of herbal medicine for COVID-19 is an effective treatment, designing herbal medicine from scratch is costly and time-consuming. In such a case, we borrowed the idea from drug repurposing to mining potential therapeutic herbal formulas by developing a collaborative filtering approach. Drug repurposing is the strategy that given drugs with a known mechanism of action and pharmacokinetics can be considered the priori knowledge of a specific domain. When the potential effects of the known drug are discovered, it makes the drug more effective and safer to be used, without having to start from scratch.

## Methods

### Molecular Docking

#### The Significance of Molecular Docking

Molecular docking is the process of mutual recognition between two or more molecules through geometrical complementarity and energy matching. Geometric complementarity is the match in shape between molecules, and energy complementarity is the analysis of the interaction forces between molecules. Molecular docking can be used to predict the binding conformation of small molecules of compounds in biological macromolecules and their binding ability, and plays an important role in the study of protein–ligand interaction mechanisms and structure-based computer-aided herbal medicine design (Khodair et al., 2021). To date, this method is still used for rapid screening of compounds from a compound library containing thousands of compounds, saving significant time and cost for herbal medicine discovery and development (Ewing and Kuntz, 2015). Therefore, in this study, the molecular docking technique is chosen to synthesize the binding conformation between molecules.

#### Scoring Functions

Common molecular docking software includes AutoDock Vina, AutoDock4, and QVina. In this study, we use QVina for molecular docking, which is a new docking tool that utilizes the powerful scoring function of AutoDock Vina and the accelerated search algorithm of QVina to increase the search space, and it has very good results for blind docking. The purpose of molecular docking is to find the best binding mode between ligand and receptor molecules (Hassan et al., 2020). Therefore, the most important problem faced is how to evaluate the binding strength between docked molecules. In this study, we combine certain advantages of knowledge potential and empirical scoring functions for derivation, extracting empirical information from conformational preferences and experimental affinity measurements of receptors and ligands, and thus deriving a scoring function for the binding affinity of herbal medicine compounds and SARS-CoV-2 proteins. Specifically, the scoring function c is calculated using the following equation (Quiroga and Villarreal, 2016):

$$c={\text{Σ}}_{i<j}f_{t_{i}t_{j}}\left(r_{ij}\right).$$ (1)

Each atom *i* is assigned a type $t_{i}$ , and each atom j is assigned a type $t_{j}$ . Then the symmetric interaction function $f_{t_{i}t_{j}}$ of the distance $r_{ij}$ between atoms is defined. Furthermore, the scoring function can be obtained from the $h_{t_{i}t_{j}}$ function of the weighted sum of spatial interactions. Functions $h_{t_{i}t_{j}}$ include all pairs of atoms with different weights, hydrophobic interactions between hydrophobic atoms, and hydrogen bonding forces.

$${\text{f}}_{{\text{t}}_{\text{i}}t_{\text{j}}}\left({\text{r}}_{\text{ij}}\right)={\text{h}}_{{\text{t}}_{\text{i}}t_{\text{j}}}\left({\text{d}}_{\text{ij}}\right),$$ (2)

$$d_{ij}=r_{ij}-R_{t_{i}}-R_{t_{j}},$$ (3)

where R_t_ is the Van der Waals radius of a t-type atom.

The spatial interaction distances for the molecular docking process can be obtained using Gaussian distances (Eqs 4, 5). If both molecules in the docking matrix are hydrophobic, it can be calculated using Eq. 6. The repulsion of molecules can be calculated using Eq. 7. If the molecule is composed of a hydrogen bond donor and a hydrogen bond acceptor, Eq. 8 is added to the calculation.

$$Gauss_{1}\left(d\right)=e^{-{\left(d/0.5Å\right)}^{2}},$$ (4)

$$Gauss_{2}\left(d\right)=e^{-{\left(d-3Å/2Å\right)}^{2}},$$ (5)

$$\text{Hydrophobic }\left(d\right)=\{\begin{array}{cc} 1, & d<0.5Å\\ 1.5Å-d, & 0.5Å\leq d\leq 1.5Å\\ 0, & d>1.5Å \end{array},$$ (6)

$$\text{Repulsion }\left(d\right)=\{\begin{array}{cc} d^{2}, & d<0\\ 0, & d\geq 0 \end{array},$$ (7)

$$\text{Hydrogen bonding }\left(d\right)=\{\begin{array}{cc} 1, & d<-0.7Å\\ \frac{d}{0.7Å}, & -0.7Å\leq d\leq 0\\ 0, & d>0 \end{array},$$ (8)

$$h_{t_{i}t_{j}}=\{\begin{array}{c} w_{1}*{\text{ Gauss }}_{1}\left(d\right)+w_{2}*\text{Gauss }\left(d\right)+w_{3}*\text{Repulsion }\left(d\right)+\\ w_{4}*\text{ Hydrophobic }\left(d\right)+\\ w_{5}*\text{ Hydrogen bonding }\left(d\right) \end{array},$$ (9)

$$R_{t}:w_{1}=-0.035579,w_{2}=-0.005156,w_{3}=0.840245,w_{4}=-0.035069,w_{5}=-0.587439,$$ (10)

After judging and calculating the aforementioned properties of the molecule, the binding of the herbal medicine compound and the SARS-CoV-2 protein is calculated using the scoring function (Eq. 9), and the coefficients in Eq. 10 are empirical constants from our experiments. When the binding free energy (binding affinity) is less than −7 kcal/ mol, we judge that the compound can effectively bind to the protein because at this time, the ligand–receptor interaction is an integrated equilibrium process and the resulting herbal medicine molecule conformation has the lowest free energy. For SARS-CoV-2 protein with small structures, the entire range is directly framed for docking, and for larger structures, the proteins are divided into multiple regions for docking separately, and then the results are manually combined.

#### SARS-CoV-2 Protein Docking Process

First, the aforementioned method is used to dock the herbal medicine and its corresponding compounds (482 herbal medicines and 13,448 compounds) obtained from the TCMSP database with the SARS-CoV-2 proteins (24 proteins) obtained from the RCSB website. We docked the herbal medicine compounds with the SARS-CoV-2 proteins 10 times each and took the average of the 10 times as the docking results. Then the molecular fingerprints and trait characteristics of each herbal medicine are embedded into a vector. Based on the embedded vector, the similarity between each of the 482 herbal medicines and each of the herbal medicine in each prescription of “three TCM prescriptions and three medicines” is calculated by collaborative filtering. Finally, based on the aforementioned “three TCM prescriptions and three medicines” ranking list, the herbal medicine with the highest similarity value is selected and formed into a prescription (i.e., herbal medicine combination). Finally, based on the ranking list of each herbal medicine in each prescription of “three TCM prescriptions and three medicines,” the one with the highest similarity value is selected and formed into a prescription (i.e., herbal medicine combination).

### Collaborative Filtering

Collaborative filtering algorithm is one of the more well-known and commonly used recommendation algorithms to predict possible herbal medicine combinations based on the docking results of molecules (Wang and Zeng, 2020). We performed the recommendation of herbal medicine combinations by the collaborative filtering algorithm and added the structural information of the compound itself to the docking values as the auxiliary information of collaborative filtering.

The training set for this task is a list of combinations of specific herbs with 482 herbs in the three-drug triad and their synergistic labels. First, given a drug combination (x, y), a molecular docking technique is used to output their respective vector representation x,y as a continuous vector representation xy. This combination vector describes how the two drugs interact through their respective biological targets. This combined vector is then docked to the new crown protein to predict its synergistic effects based on the Bliss score (Bliss, 1939). We used the Bliss score (Bliss, 1939) to predict the synergistic effect of a drug combination. Assuming that drugs x and y interact with neo-crown proteins as a result of p_x_ and p_y_, then the result of the reaction of drug combination (x, y) with neo-crown proteins is defined as

$$e_{xy}=p_{x}+p_{y}-p_{x}p_{y}.$$ (11)

A drug combination is determined to be synergistic if its actual activity p_xy_ > e_xy_. Therefore, we define its synergistic score as (Jin et al., 2021)

$$s_{xy}=p_{xy}-e_{xy}=p_{xy}-p_{x}-p_{y}+p_{x}p_{y}.$$ (12)

#### Molecular Fingerprint Calculation

Two methods, namely, MACCS and RDK, are chosen to extract the structural features of the compounds by molecular fingerprinting. The RDKit algorithm (Coley et al., 2019) is used to check the substructures between minpath and maxpath, and a total of 166 substructures are checked, with a value of 1 if the molecule has substructure and 0 otherwise, and then the substructures are hashed. We also consider three features, namely, atomic type, aromaticity, and type of bond. Under the premise of satisfying the aforementioned conditions, the length of the generated molecular fingerprint is guaranteed to be constant at 2048, and the molecular fingerprints of all the compounds included are accumulated in terms of herbal medicine. The resulting molecular fingerprints are used as the molecular fingerprints of herbal medicine.

#### Efficacy Characteristics

The efficacy, pharmacological properties, and meridians of the aforementioned herbal medicine are filtered from the herbnet database of the Institute of Herbal Medicine Information of the Chinese Academy of Traditional herbal medicine, and the information is standardized and transformed into vectors of specific length. The length of the vector is 739 dimensions, of which 40 dimensions are for medicinal properties, 14 dimensions are for meridians, and 685 dimensions are for efficacy. Finally, the vector is converted into a one-hot vector by assigning a value of 1 if the herbal medicine contains features and 0 otherwise.

#### Similarity Calculation

The similarity between herbal medicines is calculated by molecular fingerprints and efficacy traits of herbal medicines. First, the molecular fingerprints of herbal medicines are converted into one-hot vectors and spliced with efficacy shape features, and then tanimoto similarity calculation is performed from which the similarity matrix of herbal medicines is obtained. The tanimoto coefficient can be used to determine the degree of correlation between two herbal medicines.

$$Tanimato\left(X,Y\right)=\frac{X\cap Y}{X\cup Y},$$ (13)

$$T\left(x,y\right)=\frac{xy}{{\left\|x\right\|}^{2}+{\left\|y\right\|}^{2}-xy},$$ (14)

where X and Y represent the vectors composed of molecular fingerprints and efficacy traits of herbal medicine, respectively. Here, x and y are denoted as two vectors, and each element in the set is denoted as a dimension in the vector. In each dimension, the value taken is usually a value between [0, 1], xy denotes the vector product, and ||x||^2 denotes the mode of the vector, that is,

$$∥\text{x}{∥}^{2}=\sqrt{\overset{\text{n}}{\underset{\text{i}=1}{\sum }}{\text{x}}_{\text{i}}^{2}}.$$ (15)

Then, the similarity among 482 herbal medicines, three TCM prescriptions, and three medicines is calculated, mainly by calculating the similarity between these 482 herbal medicines and each herbal medicine in the formula (i.e., the value corresponding to the herbal medicines–herbal medicines similarity matrix), and the similarity value between the herbal medicine. The formula is obtained by weighting the herbal medicine contained in the formula by the position of the ruler, subject, and coordinator.

#### Similarity Calculation of Three TCM Prescriptions and Three Medicines

The similarity between the 482 herbal medicines and the herbal medicines in three TCM prescriptions and three medicines is calculated, and the top 20 herbal medicines with the highest similarity in each Lianhua Qingwen capsule are taken to form a 12 × 20 similar herbal medicine matrix, and the proportion of the herbal medicine in this matrix in the guideline-recommended TCM is calculated.

## Experimental Results

### Overview of TCM

The 482 TCMs obtained from the TCMSP database contained a total of 13,448 compounds, of which 3,243 compounds appeared in multiple TCMs, with beta-sitosterol appearing most frequently in 237 herbal medicines, followed by palmitic acid and Sitogluside which appeared in 227 and 180 herbal medicines, respectively. After absorption, distribution, metabolism, and excretion (ADME) screening, 1665 compounds remained, of which 342 compounds are found in multiple herbal medicines, with beta-sitosterol occurring most frequently in 237 herbal medicines, followed by quercetin and sitosterol, appearing in 178 and 156 herbal medicines, respectively.

### Overview of Three TCM Prescriptions and Three Medicines

There are 49 herbal medicines in the triad of three TCM prescriptions and three medicines, of which 17 herbal medicines appear in multiple formulations, with ephedra, bitter almond, and licorice appearing the most frequently with a total of five occurrences, followed by patchouli with four occurrences. These 49 herbal medicines contain a total of 3289 compounds, of which 760 compounds appeared in multiple herbal medicines, with the most frequent being palmitic acid, which appeared in 29 herbal medicines, followed by beta-sitosterol and CAM, which appeared in 21 and 20 herbal medicines, respectively. After ADME screening, 49 herbal medicines also contain 409 compounds, of which 62 compounds appear in multiple herbal medicines, with the highest number of occurrences being beta-sitosterol in 21 herbal medicines, followed by stigmasterol and kaempferol in 18 herbal medicines, respectively.

### Docking Results of All Compounds

When the compounds are docked to the herbal medicine results without ADME screening, the average of free energy values for 24 proteins is found to be below −7 kcal/mol for 3151 compounds, with chelidimerine having the lowest result of −10.73 kcal/mol. This compound obtained the lowest free energy value in 12 proteins (S, M, N, nsp2, nsp3, nsp6, nsp9, nsp10, nsp12, nsp13, nsp14, and orf6) and is also low in the other 12 proteins, which is present in the Chinese herb C. alba. The next highest free energy is neo-przewaquinone-a with a mean free energy of −10.21 kcal/mol, which is present in the TCM Salvia miltiorrhiza.

After screening by ADME, there are 388 compounds below −7 kcal/mol, of which (2aR,2′R,4R,6aR,6bS,8aS,8bR,11aS,12aR,12bR)-4-((S)-2-(2,6-dimethylphenyl) propoxy)-5′,5′,6a,8a-tetramethyl-8-methylenedocosahydro-1Hspiro[pentaleno[2,1-a]phenanthrene-10,2′-pyran] has the lowest free energy value of −9.29 kcal/mol and is present in the TCM Puncturevine Caltrop Fruit. It is followed by bisindigotin with a free energy of -9.24 kcal/mol, which is present in the TCM indigoplant and natural indigo.

### Docking Results of Compounds in Three TCM Prescriptions and Three Medicines

Without ADME screening, the results of compound-herbal medicine docking in three TCM prescriptions and three medicines in average over 24 protein free energy values show that 548 compounds are below −7 kcal/mol, with the lowest result of −10.21 kcal/mol for neo-przewaquinone-a. The compound obtained the lowest free energy value in seven proteins (E, nsp4, nsp7, nsp8, orf7a, orf8, and orf10) and is found in the herbal medicine *Salvia divinorum*. It is followed by Xambioona with a mean free energy of −8.89 kcal/mol, which is present in the herbal medicine licorice. The top ten compounds with the lowest free energy without ADME screening are shown in Table 2.

**TABLE 2:** Top 10 compounds without ADME screening.

Index	Mol	Compound	Molecular structure	Free energy	Major presence of herb
1	MOL001479	Chelidimerine	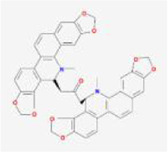	−10.73	Greater Celandine Herb
2	MOL007062	Neo-przewaquinone a	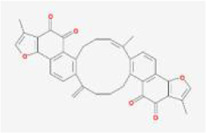	−10.21	Salvia miltiorrhiza
3	MOL004227	Sanguidimerine	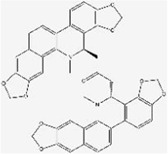	−9.71	Corydalis Corydalis
4	MOL005085	Chelidimerine	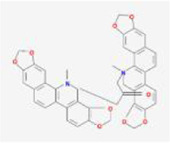	−9.55	Shinyleaf Pricklyash Root
5	MOL012727	Mulberrofuran K	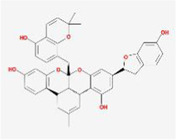	−9.4	White Mulberry Root-bark
6	MOL012728	Mulberrofuran M	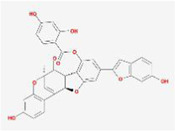	−9.34	White Mulberry Root-bark
7	MOL008559	(2aR,2′R,4R,6aR,6bS,8aS,8bR,11aS,12aR,12bR)-4-((S)-2-(2,6-dimethylphenyl)propoxy)-5′,5′,6a,8a-tetramethyl-8-methylenedocosahydro-1H-spiro[pentaleno[2,1-a]phenanthrene-10,2′-pyran]	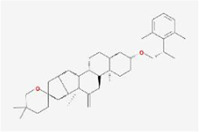	−9.29	Puncturevine Caltrop Fruit
8	MOL002041	C-Curarine	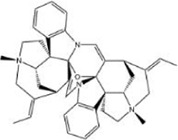	−9.27	Chinese Arborvitae Twig
9	MOL011100	Bisindigotin	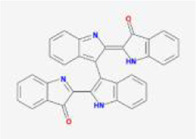	−9.24	Indigoplant Natural Indigo
10	MOL007238	Physalinb	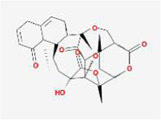	−9.21	Franchet Groundcherry Calyx and Fruit

After ADME screening, 90 compounds are found to be below −7 kcal/mol, with compound Xambioona having the lowest free energy value. The next compound is 6-(3-oxoindolin-2-ylidene)indolo[2,1-b]quinazolin-12-one with a mean free energy of −8.51 kcal/mol, which is present in the herbal medicines Panax quinquefolium, Da Qing Ye, and Qing Dai.

#### Docking Results for Herbal Medicine

In six separate ways, the results of the top ten are summarized in Table 3.

**TABLE 3:** Molecular docking results of six different ways of herbal medicine.

Strategy	Original value + ALL	Original value + AD	Second classification + ALL	Second classification + ADME	Relu + all	Relu + ADME
1	*Chrysanthemum*	Liquoric Root	Liquoric Root	Liquoric Root	Liquoric Root	Liquoric Root
2	Chinese Thorowax Root	Lucid Ganoderma	Chinese Rose Flower	Lucid Ganoderma	Chinese Rose Flower	Lucid Ganoderma
3	Liquoric Root	Lightyellow *Sophora* Root	Fiveleaf Gynostemma Herb	Salvia miltiorrhiza	Fiveleaf Gynostemma Herb	Chinese Rose Flower
4	Ephedra Herb	Barbary Wolfberry Fruit	*Achyranthes* root	Chinese Rose Flower	Largetrifoliolious Bugbane Rhizome	Salvia miltiorrhiza
5	Myrrh	Myrrh	Largetrifoliolious Bugbane Rhizome	Corydalis Corydalis	*Achyranthes* root	Corydalis Corydalis
6	Loquat Leaf	Baical Skullcap Root	Lucid Ganoderma	Corynoline	Lucid Ganoderma	Corynoline
7	Perilla Fruit	Gambir Plant	Loquat Leaf	Chinese Date	Loquat Leaf	Chinese Date
8	Ginkgo Leaf	Amur Corktree Bark	Myrrh	Myrrh	*Ilex* latifolia Thunb	Amur Corktree Bark
9	Lucid Ganoderma	Salvia miltiorrhiza	*Ilex* latifolia Thunb	Lightyellow *Sophora* Root	Ardisia Herb	Greater Celandine Herb
10		Barbed Skullcap Herb	Ardisia Herb	Greater Celandine Herb	Myrrh	Myrrh

#### Docking Results of Three TCM Prescriptions and Three Medicines

According to the vector value of herbal medicines and the situation of different herbal medicines in the prescription, the weighted average of three TCM prescriptions and three medicines is calculated, respectively. When not weighted according to the composing principle, Jinhua Qinggan granules have the highest vector value regardless of the way they are ranked, followed by the Lianhua Qinggan capsule and Xuebijing. According to the composing principle, Jinhua Qinggan granules have the highest docking value when sorted in three ways. The specific sorting results are shown in Table 4.

**TABLE 4:** Ranking of docking results of three TCM prescriptions and three medicines.

Sort	Calculation method	Original value + ALL	Original value + ALL	Second classification + ALL	Second classification + ALL	Relu + all	Relu + ADME
1	Original value	Jinhua Qinggan Granule	Jinhua Qinggan Granule	Jinhua Qinggan Granule	Jinhua Qinggan Granule	Jinhua Qinggan Granule	Jinhua Qinggan Granule
2		Xuebijing	Lianhua Qingwen capsule	Lianhua Qingwen capsule	Lianhua Qingwen capsule	Lianhua Qingwen capsule	Lianhua Qingwen capsule
3		Lianhua Qingwen capsule	Xuanfei Baidu recipe	Xuebijing	Xuanfei Baidu recipe	Xuebijing	Xuanfei Baidu recipe
4		Qingfei Baidu Decoction	Qingfei Baidu Decoction	Xuanfei Baidu recipe	Xuebijing	Xuanfei Baidu recipe	Xuebijing
5		Huashi Baidu recipe	Huashi Baidu recipe	Huashi Baidu recipe	Huashi Baidu recipe	Huashi Baidu recipe	Huashi Baidu recipe
6		Xuanfei Baidu recipe	Xuebijing	Qingfei Baidu Decoction	Qingfei Baidu Decoction	Qingfei Baidu Decoction	Qingfei Baidu Decoction
1	Composing principle weighted value	Xuebijing	Jinhua Qinggan Granule	Huashi Baidu recipe	Huashi Baidu recipe	Jinhua Qinggan Granule	Jinhua Qinggan Granule
2		Jinhua Qinggan Granule	Xuebijing	Qingfei Baidu Decoction	Qingfei Baidu Decoction	Lianhua Qingwen capsule	Xuanfei Baidu recipe
3		Lianhua Qingwen capsule	Xuanfei Baidu recipe	Xuanfei Baidu recipe	Lianhua Qingwen capsule	Xuebijing	Xuebijing
4		Qingfei Baidu Decoction	Lianhua Qingwen capsule	Xuebijing	Xuebijing	Xuanfei Baidu recipe	Lianhua Qingwen capsule
5		Huashi Baidu recipe	Qingfei Baidu Decoction	Lianhua Qingwen capsule	Xuanfei Baidu recipe	Qingfei Baidu Decoction	Qingfei Baidu Decoction
6		Xuanfei Baidu recipe	Huashi Baidu recipe	Jinhua Qinggan Granule	Jinhua Qinggan Granule	Huashi Baidu recipe	Huashi Baidu recipe

#### Diagram of Docking Results of TCM Compounds

**FIGURE 2:**
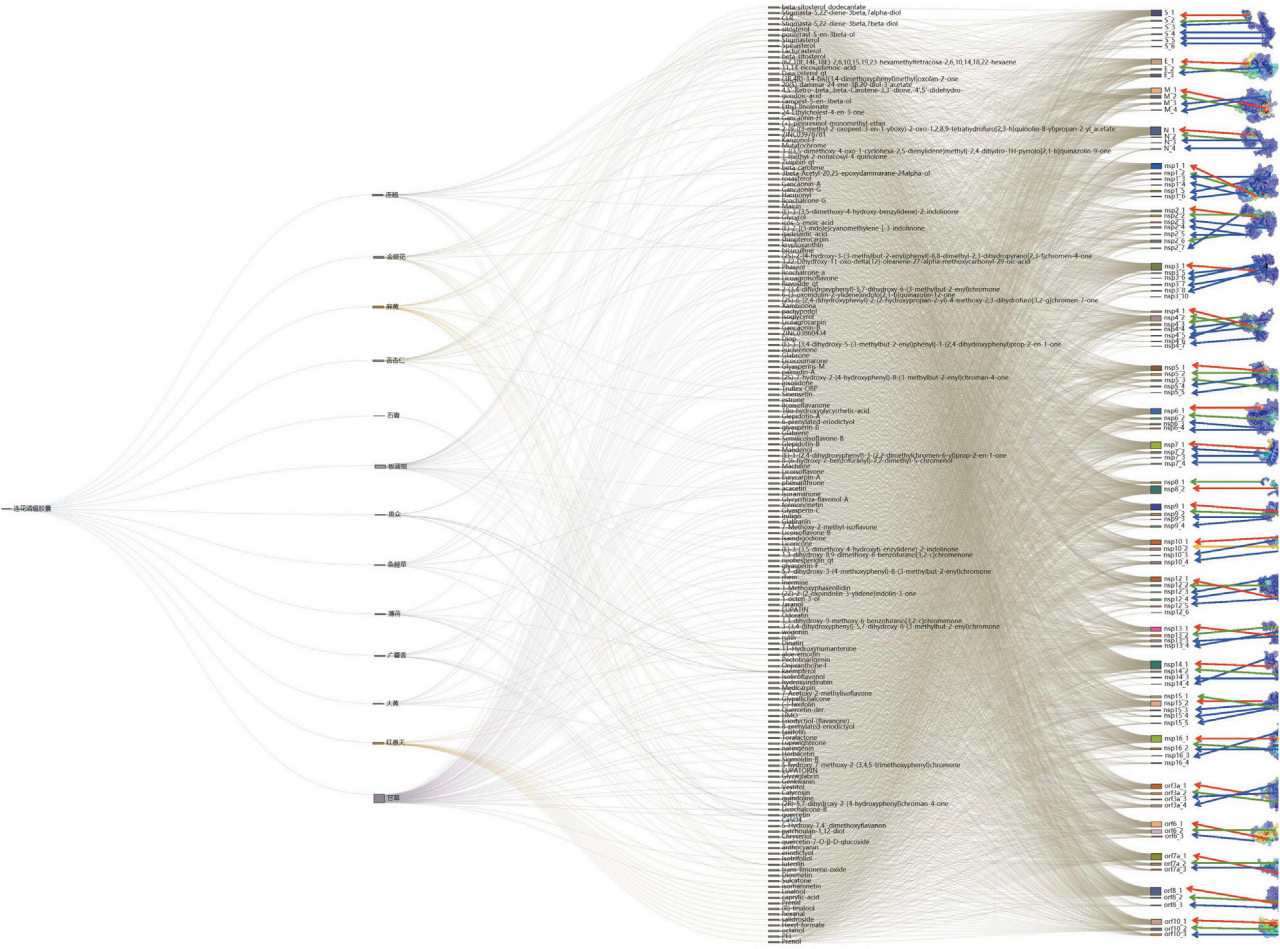
Diagram of docking results of herbal medicine compounds.

The distance −7 kcal/mol is used as the criterion for whether the receptor protein is bound to the compound. The binding position of the compound and the protein is determined, and the docking sites of the compound on the protein are divided. The results are shown in Figure 2.

#### Collaborative Filtering Results

The results of collaborative filtration of herbal medicine in Xuebijing are shown in Table 5. The yellow mark is the herbal medicine in Xuebijing, and each column is arranged from high to low according to the similarity, and the red mark is the herbal medicine appearing in the guide. The principle herbal medicine saxifrage has the highest similarity with safflower. Among the 20 herbal medicines with the highest similarity, six are in the SARS-CoV-2–recommended medicines, among which the highest similarity is in the model of winter flowers, followed by ginseng, mulberry leaf, semen, Chuanxiong, angelica, and yam. The herbal medicines with the highest similarity to the subject medicines Chuanxiong and Radix Paeoniae are Tiannanxing and Qianhu, and the first 20 herbal medicines have eight and two herbal medicines in the SARS-CoV-2–recommended herbal medicines, respectively. The herbal medicines with the highest similarity to the adjuvant medicine Dan Shen and Angelicae are Tianzhu Huang and Mang Jiaozi, and five and six herbal medicines are in the SARS-CoV-2–recommended herbal medicine, respectively.

**TABLE 5:** Results of collaborative filtration of herbal medicine in Xuebijing.

Index	Red flowers	Chuanxiong	Dan ginseng	Angelica	Red peony
1	Sand spines	Tiannan star	Tianzhu yellow	Before hu	Man jing zi
2	Winter flowers	Red flowers	Good mother grass	*Caenorhabditis elegans*	Summer withered grass
3	Pepper	Angelica	Tan Xiang	Rose	Qian grass
4	Ginseng	Winter flowers	Mulberry leaves	Chuanxiong	Bone broken fill
5	Tiannan star	Chuan Bei mother	Conceptual	Malts	*Artemia*
6	Jujube	Lei Gong rattan	Chicken excrement rattan	Chuan Bei mother	Lychee core
7	Prevent yourself	Before hu	In front of the car	Jujube	Dragon bile
8	Mulberry leaves	Black beans	Cicc	Red flowers	Chicken vine
9	Mab	Burdock	Yam	Ginseng	Xin Yi
10	Before hu	Malts	Guan Huang Bai	The dogwood	Golden buckwheat
11	Half summer	Chai Hu	Red peony	Sand kernel	Lily
12	Chuan Bei mother	Pepper	Chun skin	Wu dogwood	West red flowers
13	Black beans	Half summer	Rohan fruit	Purple Su Zi	Short tea
14	Acid jujube kernel	Piper seal	Chai Hu	Pepper	Ginkgo leaves
15	Chuanxiong	Sand spines	Soil tuckahoe	Stemon white	No medicine
16	Burdock	*Lactobacillus*	Lei Gong rattan	Big green leaves	Citron
17	Angelica	Mulberry leaves	Black sesame seeds	Mung bean	White peony
18	Malts	Amarlane	Pour buckle grass	Quinb	Huang Lian
19	Orange stalk	Chinese wolfberry	Secret flowers	Cardamom	Guangxi branch
20	Yam	Calcutrix	The green seal	Ma Huang	Pour buckle grass

The top 20 herbal medicines with the highest degree of similarity between the 12 herbal medicines of Lianhua Qingwen capsule and the 482 herbal medicines docking are shown in Supplementary Table S1. A total of 90 herbal medicines are recommended in the eighth edition of COVID-19 guidelines. The herbal medicine with the highest similarity with Honeysuckle flower in the Lianhua Qingwen capsule is Humulus. Eight of the top 20 herbal medicines are among the recommended herbal medicine of COVID-19, including Weeping Forsythia, Dahurian Patrinia Herb, Common Coltsfoot Flower, Perilla Fruit, Common Anemarrhena Rhizome, Golden Thread, and Great Burdock Achene. Chinese Magnoliavine Fruit is the herbal medicine with the highest similarity to principal herbal medicine, and eight of the top 20 herbal medicines are also among the recommended herbal medicines of COVID-19, including Heartleaf Houttuynia Herb, Golden Thread, Baizhi, Honeysuckle Flower, European Verbena, Immature Bitter Orange, *Cassia* Twig, and Fortune Eupatorium Herb. The herbal medicines with the highest similarity in the ministerial medicines Bitter Apricot Seed, Gypsum, and Ephedra Herb are Willowleaf Swallowwort Rhizome, Chinese Gall, and Perilla Fruit, respectively, with a total of 13 herbal medicines in the guide. The ministerial medicines Rhubarb, Cablin Potchouli Herb, Peppermint, Cyrtomium Rhizome, Rhodiola, Indigowoad Root, and Heartleaf Houttuynia Herb have the highest similarity, including Chinaberry Bark and Root-bark, Storax, Wild *Chrysanthemum*, Java Brucea Fruit, Common Selfheal Fruit-Spike, Indigoplant, and Airpotato Yam Rhizome. A total of 24 herbal medicines are recommended by COVID-19.

The top 20 herbal medicines with the highest degree of similarity between the 12 herbal medicines of Xuanfei Baidu prescription and the 482 herbal medicines docking are shown in Supplementary Table S2. Six of the twenty herbal medicines with the highest docking similarity to Ephedra are in the SARS-CoV-2 herbal medicine recommendation guide, among which Zhi Mu has the highest similarity to Ephedra, followed by Angelica sinensis, Sheng Jiang, Chuan Xiong, Da Qing Ye, and Hong Hua. Five herbal medicines with the highest similarity to bitter almond, scaphozel, and bitter almond docking, respectively, appeared in the top 20 herbal medicines recommended by SARS-CoV-2, and the highest herbal medicines appearing in the SARS-CoV-2–recommended herbal medicine are Mu Xiang, Suhe Xiang, and Zhi Mu. The top 20 herbal medicines with the similarity of gypsum did not appear in the SARS-CoV-2–recommended herbal medicine.

The top 20 herbal medicines with the highest degree of similarity between the 20 herbal medicines of Qingfei Baidu decoction and 482 herbal medicines docking are shown in Supplementary Table S3. Among the top 20 herbal medicines with the highest docking similarity of Scutellaria baicalensis, Ginger, and Pinellia, eight herbal medicines appeared in the recommended herbal medicines of COVID-19, and the herbal medicines with the highest similarity are Coptis chinensis, Poria cocos, and *Astragalus* membranaceus. This is followed by Ephedra, Radix et Rhizoma, Zedoary, and Yam, with six of the top 20 herbal medicines with the highest docking similarity appearing in the SARS-CoV-2–recommended herbal medicine.

The top 20 herbal medicines with the highest degree of similarity between the 11 herbal medicines of Jinhua Qinggan granule and 482 herbal medicines docking are shown in Supplementary Table S4. The top 20 herbal medicines with the highest docking similarity to Scutellaria baicalensis and Zhi Mu are the most abundant among the SARS-CoV-2–recommended herbal medicines. The herbal medicine with the highest similarity to Scutellaria baicalensis is Huanglian, which is the SARS-CoV-2–recommended herbal medicine. The herbal medicine with the highest similarity to Zhi Mu is Polygonum officinale, and the next highest herbal medicine is Da Qing Ye, which is a SARS-CoV-2–recommended herbal medicine. Forsythia, honeysuckle, and burdock had seven herbal medicines with top 20 similarities in the SARS-CoV-2–recommended herbal medicines, respectively. The herbal medicine with the highest similarity in forsythia is northern schisandra, and the herbal medicine with the highest ranking in the SARS-CoV-2–recommended herbal medicine is ichthyopodium. The herbal medicine with the highest similarity to Honeysuckle is Humulus lupulus, and the herbal medicine with the highest similarity in SARS-CoV-2–recommended herbal medicine is Forsythia. The herbal medicine with the highest similarity to Burdock is Sandalwood, which also appears in the SARS-CoV-2–recommended herbal medicines.

The top 20 herbal medicines with the highest degree of similarity between the 13 herbal medicines of Huashi Baidu prescription and the 482 herbal medicines docking are shown in Supplementary Table S5. The top 20 herbal medicines with the highest docking similarity to Semen Astragali are the most numerous among the SARS-CoV-2–recommended herbal medicines, with a total of eight herbal medicines. The top two herbal medicines with the highest similarity to Semen Astragali and Sandalwood are both in the SARS-CoV-2–recommended herbal medicine. The next most similar herbal medicine is Ephedra, whose highest similarity is Ziziphi, and the most similar herbal medicine in the SARS-CoV-2–recommended herbal medicine is Zhi Mu. Bitter almond, patchouli, scape seed, and *Astragalus* are among the top 20 herbal medicines with the highest similarity, and five of them appear in the SARS-CoV-2–recommended herbal medicine. The herbal medicine with the highest similarity are Bai Qian, Suhe Xiang, mustard seed, and Han Xia, and the herbal medicine that appeared in the SARS-CoV-2–recommended herbal medicine with the highest similarity are Mu Xiang, Suhe Xiang, Zhi Mu, and Han Xia.

## Evaluation

Qingfei Baidu decoction, the lung soup, is suitable for light, normal, and heavy patients; Xuanfei Baidu prescription is suitable for normal type of dampness and toxin stagnation of the lung evidence; and Huashi Baidu prescription is suitable for heavy type of epidemic and toxin occlusion of the lung evidence. Lianhua Qingwen Capsule and Jinhua Qinggan granule are indicated for clinical symptoms of malaise with fever during the medical observation period, while Xuebijing is indicated for heavy- and critical-type patients during the clinical treatment period. Our recommended drug combinations can be used and adjusted according to the different clinical manifestations and the actual situation of the patients.

### Xuebijing-Recommended Formulas

Xuebijing-recommended Eq. 1: sea buckthorn, tiannanxing, geranium, antebellum, and bramble seed. Tiannanxing: It is a warming and cold phlegm medicine, with the function of dispelling wind and stopping spasm, warming, and resolving cold phlegm; Tianzhu Huang: clearing heat and resolving phlegm, cooling the heart, and relieving fright; Qianhu: dispersing wind-heat, lowering qi, and resolving phlegm; and Manchuria: dispersing wind-heat and clearing the head and eyes. In summary, our recommended formula can also be used in severe and critical cases of novel coronavirus pneumonia, especially for patients with heavy cough and phlegm.

Xuebijing-recommended Eq. 2: Zanthoxylum, safflower, motherwort, Humulus, and Xia Kuo Cao. Safflower: moisten the lung and lower the Qi, resolve phlegm, and relieve cough; safflower: invigorate blood circulation, dispel blood stasis, and relieve pain; motherwort: invigorate blood circulation, regulate menstruation, diuretic, reduce swelling, clear heat, and detoxify; Xia Ku Cao: antibacterial action; and Humulus: clear heat and detoxify, diuretic, and laxative; in summary, the recommended formula II is suitable for patients with heavy or critical forms of novel coronavirus pneumonia, or with underlying diseases such as coronary heart disease.

### Lianhua Qingwen Capsule–Recommended Formulas

The recommended formula of Lianhua Qingwen Capsule 1: North Schisandra, Humulus, Bai Qian, Wu Bei Zi, Perilla, Neem Bark, Suhe Xiang, Wild *Chrysanthemum*, Crow’s Nest, White Hair Xia Kuo Cao, Polygonum macrophyllum, Huang Yao Zi, and Perilla (repeated drugs will not be repeated). Humulus: clearing heat and detoxifying, diuretic, and laxative; neem bark: killing insects and healing ringworm; Suhe Xiang: opening the orifice to remove obscenity, opening up phlegm, moving Qi, and relieving pain; Crow’s bile: clearing heat, detoxifying, killing insects, and intercepting malaria; white hair Xia Gu Cao: clearing heat and detoxifying, resolving phlegm and relieving cough, cooling blood, and dispersing blood; and yellow herb seed: dispersing knots and eliminating galls, clearing heat and detoxifying, cooling blood, and stopping bleeding.

The recommended formula of Lianhua Qingwen Capsule 2: Gonglao wood, Radix Codonopsis pilosulae, Tianshan snow lotus, Corrugated seeds, Moxa, Mugwort leaf, Semen lily of the valley, Lungwort, Mallow leaf, Big green leaf, Sansho root, and Coriander. Muxiang: Promotes the flow of Qi and relieves pain, regulates the middle, and directs stagnation; Mugwort: warms the menstruation and stops bleeding, disperses cold and pain, dispels dampness, and relieves itching; Lungwort: clears heat and detoxifies the blood, activates blood circulation, and reduces swelling; Malvaceae: clears heat and detoxifies the blood, relieves phlegm, diuresis, and laxative; and Sander root: clears heat and detoxifies the blood, disperses blood stasis, and relieves pain.

### Xuanfei Baidu Prescription–Recommended Formulas

The effects of each drug in the original formula of Xuanfei Baidu prescription include dispelling phlegm, and relieving cough and asthma; moistening the intestines; lowering Qi, opening paralysis, drying dampness, and strengthening the spleen; dispelling wind and dampness; brightening the eyes, activating blood circulation, and dispersing blood stasis; dispelling wind and clearing ligaments; clearing heat and dampness; detoxifying sweating and relieving symptoms; promoting the lung; cooling the blood and stopping bleeding; clearing heat and generating fluid; diuresis and laxative; relieving summer heat; removing steam; aromatizing dampness; harmonizing the stomach and stopping vomiting; dispelling summer heat and relieving toxins; activating blood and clearing menstruation; promoting water retention and decongesting swelling; intercepting malaria, benefiting Qi, and tonifying the middle; relieving pain; moistening the lung relieving cough; diaphoretic and detoxifying; harmonizing various herbs, resolving phlegm and Qi; eliminating food, dipping the lung, and lowering Qi; promoting water retention and swelling; expelling evil and clearing heat from the muscle; and relieving irritation and thirst.

The first group of recommended drug combinations are snake bed seed, white front, chicory, perilla seed, yucca, Xia Gu Cao, Suhe Xiang, perilla, and mustard seed, with five times the seed. Among them, the snake bed seeds and mustard seeds dispel wind, Bai Qian and perilla seeds lower qi and relieve cough, chicory and Xia Gu Cao clear heat, Su He Xiang and Pei Lan aromatize dampness, and Wu Bei Zi is an astringent to the lung; the drugs in Xuanfei Baidu prescription also have these effects, and the two formulas have similar efficacy.

The second recommended combination of drugs is one branch yellow flower, Tianshan snow lotus, hooked vine, shepherd’s purse, coix seeds, Chuan Shao Gan, mugwort leaf, stretching grass, coriander, peony seeds, polygonum grandiflorum, and corrugated seeds. A branch of yellow flower, hooked vine, shepherd’s purse, and coix seeds clear heat; Tianshan snow lotus dispels wind; mud bramble calms asthma; mugwort and elongated tendon grass dispel dampness; coriander detoxifies; and corrugated seeds dissolve phlegm. All these recommended drugs are consistent with the effects in the formula.

### Qingfei Baidu Decoction–Recommended Formulas

The effects of each drug in the original formula of Qingfei Baidu decoction include diuresis and dampness, dispersing cold and relieving symptoms, warming the meridians, promoting the flow of Yang and Qi, dispelling phlegm and relieving cough, calming asthma, moistening the intestines, lowering Qi and opening paralysis, permeating dampness and water, strengthening the spleen and stomach, tranquilizing the mind and tranquilizing the spirit, generating sweat and relieving symptoms, promoting the lung and relieving asthma, relieving symptoms and fever, relieving the liver and depression, raising Yang Qi, promoting water and swelling, strengthening the spleen and Qi, drying dampness and water, stopping sweating, aromatizing dampness in the fetus, relieving stomach and vomiting, dispelling summer heat and relieving symptoms, clearing heat and fire, drying dampness and detoxifying the blood, stopping bleeding, moistening the lung, and lowering Qi in the fetus. It is used to relieve phlegm and cough, moisten the lung and lower the qi, relieve phlegm and cough, dispel cold and relieve symptoms, subdue rebelliousness and vomiting, dry dampness and phlegm, subdue rebelliousness and vomiting, eliminate lumpiness and disperse knots, induce diuresis and permeate dampness, relieve heat and promote drenching, tonify the spleen, nourish the lung, consolidate the kidney, benefit the essence, benefit the qi and tonify the middle, relieve acute pain, moisten the lung and relieve cough, relieve fire and detoxify, harmonize all medicines, lower the qi, regulate the middle, awaken the body, dispel cold and wind, relieve pain, warm the lung and dissolve drinks, open the orifices, relieve muscle and clear heat, relieve irritability and thirst, break up Qi and eliminate accumulation, and resolve phlegm and disperse phlegm.

The first group of drug combinations recommended are powdered dioscorea, pepper, Bai Qian, cat’s claw herb, Zizyphus, sericea, Suhe Xiang, Ping Bei Mu, Huang Lian, Chang Shan, Tu Fu Ling, Huang Qi, Xuan Shen, Sour date palm, Pei Lan, Atractylodes, and pepper. The drugs include powdered dioscorea, pepper, silkworm sand, Suhe Xiang, Tu Fu Ling, Pei Lan, Atractylodes, pepper to benefit dampness, Bai Qian, purple sage to lower qi, cat’s claw herb, Ping Bei Mo, Chang Shan to resolve phlegm, Huang Lian, Xuan Shen to clear heat, *Astragalus* to strengthen the spleen and benefit qi, and sour date palm to tranquilize the mind, and the original formula has the same effect.

The second group of recommended drug combinations is Panax ginseng flower, Ganoderma lucidum, Tianshan snow lotus, white ganoderma, cape berry, chicken bone herb, mugwort leaf, cornus, northern schisandra, earth maidenha, goldenrod, dried ginger, sandalwood, earth poria, black sesame, coriander, sea gold sand, ganoderma lucidum, and manzanita. Among these medicines, Panax ginseng flower, white creeper, shepherd’s purse, chicken bone herb, and earthen berries clear heat; Ganoderma lucidum benefits qi; Tianshan snow lotus dispels wind; mugwort leaf, corn mullein, Tu Fu Ling, and Hai Jin Sha dispel dampness; northern schisandra, dry ginger, and Ganoderma lucidum strengthen the spleen; Yang Jin Hua and Manshan Hong stop cough; sandalwood stops pain; black sesame benefits essence; and coriander detoxifies toxins. The recommended drugs have the same efficacy as the original formula.

### Jinhua Qinggan Granule–Recommended Formulas

Jinhua Qinggan granule–recommended Eq. 1: five times the seeds, white before, the northern five-flavored seeds, perilla seeds, sandalwood, summer grass, humulus, Huang Lian, wild chrysanthemum, polygonum big green leaf, Celosia, and pelargonium. Wu Bei Zi: Relieve lung and fire, reduce phlegm and drink, relieve cough, dispel the edge, night sweats, vomiting, blood loss, children's night crying symptoms, cure red eyes and dampness, reduce swelling toxin, throat numbness, ulcers, gold sores; Wu Wei Zi: promotes meridians; strengthens the spleen, and opens the stomach; Sandalwood can regulate the Wei Qi and regulate beer lung, and benefit the chest and diaphragm; Xia Ku Cao: tuberculosis mice with mild reduction in tuberculosis index, lung lesions slightly reduced; Huang Lian: clearing heat and drying dampness, dipping fire and detoxicating. It has a significant inhibitory effect on both positive and negative bacteria *in vitro*; Daphyllum: clearing heat and detoxifying, cooling blood, and eliminating blemishes; Phellodendron: treating summer exopathogenic, fever, headache, generalized bone pain, stabbing pain in both eyes, chest tightness and nausea, and irregular bowel movement. In conclusion, this recommended formula is suitable for both light and common types of novel coronavirus pneumonia.

Jinhua Qinggan granule–recommended Eq. 2: corrugated seed, Tianshan snow lotus, GongLao wood, Capsella, Qianhu, ChuanShuGan, DangShen, BeiWuYiZi, Half branch lotus, Da QingYe, *Tribulus terrestris*, and coriander. Daphyllanthus: clearing heat and removing toxins, cooling the blood, and eliminating blemishes; semen lotus: clearing heat and removing toxins, dispersing blood stasis and stopping bleeding, diuretic, and decongesting; *Tribulus terrestris*: calming the liver, relieving depression, dispelling wind, and brightening the eyes; coriander: publishing and penetrating rashes, eliminating food and appetite, relieving pain, and detoxifying. In summary, the recommended formula is suitable for patients with novel coronavirus, mild pneumonia, common type, or with hypertensive underlying disease and concurrent Qi deficiency.

### Huashi Baidu Prescription–Recommended Formulas

The effects of each drug in the original formula of Huashi Baidu prescription include lowering rebelliousness and stopping vomiting; dispersing lumpiness and dissipating knots, drying dampness and warming the middle class, dispelling phlegm and intercepting malaria, benefiting Qi, and tonifying the middle class; relieving urgency and pain; moistening the lung and stopping cough; dipping fire and detoxicating; harmonizing the herbs, dipping the lung, and lowering Qi; dispelling phlegm and calming asthma; inducing diuresis and subduing swelling; expelling and expelling evil, tonifying Qi, and fixing the surface; promoting diuresis; promoting toxicity and draining pus; astringing sores, clearing heat, and cooling the blood; invigorating blood, removing blood stasis, and relieving heat in the muscles; and relieving irritability and thirst.

The recommended first group of drug combinations includes snake’s bed sedge, white foreskin, cat’s claw herb, red ginseng, perilla seed, neem bark, sulforaphane, *Astragalus*, persimmon tip, pelargonium, mustard seed, half summer, bramble seed, and fivefold seed. Among them, cat’s claw herb and halfsia can dissolve phlegm; red ginseng and *Astragalus* strengthen the spleen; neem bark, mustard seed, and bramble seed dispel wind; Suhexiang and pelargonium aromatize and dissolve dampness; snake bed seed, bai qian, and persimmon tip lower the qi; and five-fold seed is an astringent to the lung, which are consistent with the efficacy of the original formula and can be recommended.

The second group of recommended drug combinations is one branch of yellow flower, Tianshan snow lotus, white creeper, Pu huang, shepherd’s purse, mullein, mugwort, sandalwood, atractylodes, coriander, polygonum grandiflorum, chicken bone grass, Xia Ku Cao, and corrugated seeds. Among them, one branch yellow flower, white creeper, shepherd’s purse, polygonum officinale, chicken bone herb, and summer cress clear heat; Tianshan snow lotus dispels wind; corrugated root dissolves phlegm; Pu Huang facilitates water; Mu Xiang and sandalwood move qi; mugwort and atractylodes dispel dampness; and coriander detoxifies. The efficacy of all these drugs is consistent with the efficacy of the original formula.

In summary, the recommended drugs are consistent with the efficacy of the drugs in the three triads, so the recommended prescription can be considered reasonable from the perspective of Chinese medicine.

## Data Availability

The raw data supporting the conclusions of this article will be made available by the authors, without undue reservation.

## Footnotes

1
https://www.worldometers.info/coronavirus/.

2
https://www.afro.who.int/news/expert-panel-endorses-protocol-covid-19-herbal-medicine-clinical-trials.
