# Supplementary material for: Signaling Potential Therapeutic Herbal Medicine Prescription for Treating COVID-19 by Collaborative Filtering
Source: Front Pharmacol. 2021 Dec 24;12:759479. doi: 10.3389/fphar.2021.759479 (PMC8741270; doi:10.3389/fphar.2021.759479)
Supplement: Supplementary file 1 [file DataSheet1.docx]

Supplementary Material

Introduction to Three TCM Prescriptions and Three Medicines:

Xuebijing Injection is a formula developed on the basis of 'Xuefu Zhuyu Decoction' and under the theory of 'concurrent treatment of bacteria and toxin', which is used clinically in the form of injection. It is composed of Safflower, Red Paeony Root, Szechuan Lovage Rhizome, Salvia miltiorrhiza, Chinese Angelica and other herbs. A RCT study on COVID-19 showed that compared with the control group, the treated group had higher WBC, significantly lower CRP and ESR, and significantly lower APACHE II score. In the national protocol for the treatment of COVID-19, it is recommended to be used in heavy patients with both qi and ying burnt evidence or in critical patients with internal closure and external detachment.

Lianhua Qingwen Capsule is a Chinese patent medicine developed during SARS in 2003. It is composed of Weeping Forsythiae Capsule, Honeysuckle Flower, Ephedra Herb, Bitter Apricot Seed, Gypsum, Indigowoad Root, Cyrtomium Rhizome, Heartleaf Houttuynia Herb, Cablin Potchouli Herb, Rhubarb, Peppermint, Liquoric Root and other drugs. It is used for the treatment of influenza with fever and chills, muscle aches and pains, stuffy nose and runny nose, etc. It is especially suitable for patients with mild fever and irregular bowel movements. A number of clinical studies on COVID-19 have shown that it can change the symptoms of fever, cough, and malaise in patients, and reduce the proportion of common to severe illness. The indications for the use of Lianhua Qingwen Capsules are the same as those for Jinhua Qinggan Granules in the national treatment protocol for COVID-19.

Xuanfei Baidu prescription is a prescription summarized by academician Luqi Huang's team and academician Boli zhang's team during the rescue and treatment of COVID-19. It is composed of four formulae with addition and reduction, including Maxing Shigan decoction, maxing Yigan Decoction, Qianjin Weigan Decoction, and Tingli Dazhao xiefei decoction. It consists of Ephedra Herb, Bitter Apricot Seed, Gypsum, Coix Seed, Swordlike Atractylodes Rhizome, Sweet Wormwood Herb, Giant Knotweed Rhizome, European Verbena, Reed Rhizome, Pepperweed Seed and Liquoric Root. Data from the Chinese medicine component library indicated that Giant Knotweed Rhizome and European Verbena may be active against COVID-19. It is recommended for patients with common type of dampness and toxicity with lung evidence in the treatment protocol of novel coronavirus pneumonia promulgated by the state.

Qingfei Baidu decoction is a prescription developed during COVID-19 period, based on the addition and reduction of Maxing Shigan decoction, Shegan Mahuang Decoction, Xiaochaihu Decoction, and Wuling powder. It consists of Ephedra Herb, Almond, Gypsum, Cassia Twig, Oriental Waterplantain Rhizome, Agaric, Largehead AtractylodesRhizome, Indian Buead, Chinese Thorowax Root, Baical Skullcap Root, Ginger, Tatarian Aster Root, Common Coltsfoot Flower, Blackberrykiky Rhizome, Manchurian Wildginger, Common Yam Rhizome, Immature Bitter Orange, Tangerine Peel, Wrinkled Gianthyssop Herb, and other drugs. A retrospective study on the treatment of COVID-19 showed a significant reduction in the time to fever reduction, cough improvement, and lung CT improvement in the combination group compared to the control group. It is recommended in the national protocol for the treatment of COVID-19 in patients with severe disease and closed lung disease. It is recommended to be used in all patients diagnosed at all stages of the disease according to the xzsnational protocol for the treatment of COVID-19.

Jinhua Qinggan granule is a Chinese patent medicine developed for influenza A (H1N1), which is made of two prescriptions, maxing Shigan Decoction and Yinqiao powder. Its components include Honeysuckle Flower, Gypsum, Ephedra Herb, Bitter Apricot Seed, Baical Skullcap Root, Weeping Forsythiae Capsule, Thunberg Fritillary Bulb, Common Anemarrhena Rhizome, Great Burdock Achene, Sweet Wormwood Herb, Peppermint and Liquoric Root , used for fever, red throat, nasal obstruction and runny nose caused by exogenous pathogens. Previous studies on the efficacy of Jinhua Qinggan Granules in influenza have shown that Jinhua Qinggan Granules can significantly reduce serum levels of various cytokines in patients. An RCT study on COVID-19 showed that it could significantly reduce the symptoms of fever, cough, malaise, and sputum in mild patients. In the national protocol for the treatment of COVID-19, it is recommended to be used during the medical observation period when the clinical manifestation is fever with malaise. In this study, it is found that Jinhua Qinggan Granules in ... Drugs and... Compound can play a better binding effect on the COVID-19 protein.

Huashi Baidu prescription is also a prescription summarized by academician Luqi Huang's team and academician Boli zhang's team during the rescue and treatment of COVID-19. It is composed of Ephedra Herb, Wrinkled Gianthyssop Herb, Bitter Apricot Seed, Pinellia Tuber, Officinal Magnolia Bark, Swordlike Atractylodes Rhizome, Tsaoko Amomum Fruit, Indian Buead, Membranous Milkvetch Root, Red Paeony Root, Pepperweed Seed, Rhubarb and Liquoric Root. It is recommended in the national treatment protocol for COVID-19 for heavy patients with evidence of epidemic toxicity and closed lung.

The specific results are as follows, the yellow mark is the drug in Three TCM Prescriptions and Three Medicines, and each column is arranged from high to low according to the similarity, and the red mark is the drug appearing in the guide:

**Table S1. Results of collaborative filtration of drugs in Lianhua Qingwen capsule.**

| Index | Weeping Forsythia Capsule | Honeysuckle Flower | Bitter Apricot Seed | Gypsum | Ephedra | Rhubarb | Cablin Patchouli Herb | Peppermint | Shield-fern Rhizome | Rose-Boot | Isatis Root | Heartleaf Houttuynia Herb | Liquorice Root |
| --- | --- | --- | --- | --- | --- | --- | --- | --- | --- | --- | --- | --- | --- |
| 1 | Chinese Magnoliavine Fruit | Japanhop | WillowleafRhizome | Chinese Gall | Perilla seed | Sichuan Chinaberry Bark | Storax | Chrysanthemum Flower | Java Brucea Fruit | Common Selfheal FruitSpike | Dyers Woad Leaf | Potato | Fortune Eupatorium Herb |
| 2 | Mahonia wood | Tangshen | Snow Lotus Herb with Flower | Clam Shell | Herba Capsellae Bursa-Pastoris | Common Aucklandia Root | Argy Wormwood Leaf | Barbated Skullcup Herb | Black Nightshade | Franchet Groundcherry Fruit | Dyers Woad Leaf | Vietnamese Sophora Root | Coriander |
| 3 | Heartleaf Houttuynia Herb | Chinese Globeflower Flower | SilberianFririllaryBulb | Herba Veronicae Peregrimae | Asiatic Cornelian Cherry Fruit | Common Buried Tuber | Dutohmanspipe Fruit | Dogbane Leaf | Fritillaria ussuriensis Maxim | Golden Larch Bark | Snakegourd Fruit | Pharbitis Seed | crystalline lense |
| 4 | Golden Thread | Antifeverile Dichroa Root | Gansui Root | semen gleditsiae sinensis | Orientvine Vine | Java Brucea Fruit | Dutchmanspipe Vine | golden-rod | hinese Globeflower Flower花 | CoastalGlehnia Root | Mung bean | atrina glass | Ginkgo Leaf |
| 5 | Vietnamese Sophora Root | Hogfennel Root | Tinospora Root | Flower of Hyacinth Dolichos | Puff-Ball | Chinese Starjasmine Stem | Armand Clematis Stem | Kadsura coccinea | Paniculate Bolbostemma | Shearer’s Pyrrosia Leaf | Kelp | Black Nightshade | Fragrant Solomonseal Rhizome |
| 6 | Willowleaf Rhizome | Weeping Forsythia Capsule | Fortune Eupatorium Herb | Pine nuts | Barbary Wolfberry Fruit | Fortune’s Drynaria Rhizome | Fortune Eupatorium Herb | Chinese Honeylocust Spine | atrina glass | Millettia specisoa Champ | Common Anemarrhena Rhizome | Siegesbeckia Herb | Mountain Spicy Fruit |
| 7 | Antifeverile Dichroa Root | atrina glass | Common Clubmoss Herb | melon seed | capsicum | Ash Bark | Fourleaf Ladybell Root | Longtube Groundivy Herb | Natural Indigo | Dogbane Leaf | Germinated Barley | Combined spicebush Root | Common Aucklandia Root |
| 8 | Dahurian Angelica Root | GrosvenorMomordica Fruit | Mahonia wood | Common Bletilla Tuber | Japanhop | Buckwheat Rhizome | Dried Ginger | Tokyo Violet Herb | Rangooncreeper Fruit | Bistort Rhizome | Shepherd's purse | Chinese Magnoliavine Fruit | Sichuan Chinaberry Bark |
| 9 | Honeysuckle Flower | Platycodon Root | Epimedium Herb | Radix oryzae glutinosae | Villous Amomum Fruit | Field Thistle Herb | Wooly Datchmanspipe Herb | Fruit of Hempleaf Negundo Chastertree | Chinese Thorowax Root | golden-rod | Rose Flower | Fortune Eupatorium Herb | Heterophylly Falsestarwort Root |
| 10 | European Verbena Herb | Common Coltsfoot Flower | ChinesePulsatilla Root | Pockmarked seeds | Common Anemarrhena Rhizome | Air Potato | Cape Jasmine Fruit | Melia azedarach seed | Chicory Herb | Wild Chrysanthemum Flower | Japanhop | Saussurea involucrata | Nardostachys Root |
| 11 | Air Potato | Ephedra | Pharbitis Seed | Blackend Swallowwort Root | Dyers Woad Leaf | Pummelo Peel | Medicinal Cyathula Root | Golden Larch Bark | Thunberg Fritillary Bulb | Cochinchinese Asparagus Root | Puff-Ball | Mahonia wood | Chinese Tamarisk Twig |
| 12 | SilberianFririllaryBulb | Yerba-Detaj Herb | CommonAucklandia Root | Snakegourd Root | Chinese Angelica | Tinospora Root | Biond Magnolia Flower | Coastal Glehnia Root | Lotus Leaf | Descolor Cinquefoil Herb | Silybum marianum | Willowleaf Rhizome | Fourleaf Ladybell Root |
| 13 | Gansui Root | Willowleaf Rhizome | European Verbena Herb | Cochinchina Momordica Seed | Fresh Ginger | Mountain Spicy Fruit | Japanese thistle Herb | ChineseTamarisk Twig | Chinese Holly Leaf | Kadsura coccinea | Pepperweed Seed | Seed of CoralheadPlant | Stemona Root |
| 14 | Suberect Spatholobus Stem | Common Anemarrhena Rhizome | Citron Fruit | Appendiculate Cremastra Pseudobulb | Siberian Cocklebur Fruit | Sophora Flower | Funneled Physochlaina Root | Common Chidium Fruit | Golden Thread | radix boehmeriae | Radish Seed | Datura Flower | Biond Magnolia Flower |
| 15 | ImmatureOrange Fruit | Golden Thread | Suberect Spatholobus Stem | receptaculum nelumbinis | Sichuan Lovage Rhizome | Chinese Gentian | Villous Amomum Fruit | Turmeric Root Tuber | Catclaw Buttercup Root | Persimmon Calyx | Mustard Seed | Chicory Herb | Morinda Root |
| 16 | Chinese Globeflower Flower | Meadowrue Root and Rhizome | Heartleaf Houttuynia Herb | Lotus Stamen | Hogfennel Root | Pokeberry Root | Dried Tangerine Peel | Daidaihua | Canton Lovepea Vine | Chinese Arborvitae Twig and Leaf | Gordon Euryale Seed | European Verbena Herb | India Madder Root |
| 17 | Chinese Pulsatilla Root | Glossy Privet Fruit | Radix Hedysari | Fermented Soybean | Scallion white | Southern crane louse | Willowleaf Rhizome | Citron Fruit | CowHerb Seed | Barbated Skullcup Herb | Seaweed | SilberianFririllaryBulb | Indian Trumpetflower Seed |
| 18 | Cassia Twig | Sanqi | Medicine Terminalia Fruit | Rhizome | Dyers Woad Leaf | Shrub Chastetree Fruit | Combined spicebush Root | Atractylodes Rhizome | Giant Knotweed Rhizome | pot marigold | Asiatic Cornelian Cherry Fruit | Java Brucea Fruit | Lalang Grass Rhizome |
| 19 | Seed of Coralhead Plant | Great Burdock Achene | Areca Peel | saxifrage | Rose Flower | Indian Stringbush Root | Largetrifoliolious Bugbane Rhizome | Hairyvein Agrimonia Herb | Chinese Magnoliavine Fruit | Citronella | Barbary Wolfberry Fruit | Antifeverile Dichroa Root | Franchet Groundcherry Fruit |
| 20 | Fortune Eupatorium Herb | Chinese Angelica | Stemona Root | Potentilla | Safflower | Mahonia wood | Common Aucklandia Root | Chinese Arborvitae Twig and Leaf | Gambir Plant Nod | Appendiculate CremastraPseudobulb | Pinellia Tuber | Chinese Starjasmine Stem | Wild Chrysanthemum Flower |

**Table S2. Results of collaborative filtration of drugs in Xuanfei Baidu Recipe.**

| Index | Cang art | Bitter almond | Tiger stick | Ma Huang | White MAO root | Artemia | Guanghuoxiang | Horse whip grass | licorice | Orange red | Li Zi | gypsum |
| --- | --- | --- | --- | --- | --- | --- | --- | --- | --- | --- | --- | --- |
| 1 | Snake bed | White before | Chicory | Purple Su Zi | Jade bamboo | Summer withered grass | Su Hexiang | Perrin | Perrin | Snake bed | The mustard | Five times the son |
| 2 | A yellow flower | Tianshan snow lotus | The hook rattan | shepherd's purse | Job's tears | Sichuan shot dry | Iye | Stream grass | coriander | Mujingzi | Polygonum daqing leaf | The corrugated |
| 3 | Wild chrysanthemum | Ibel mother | Chai Hu | The dogwood | The hemp yellow root | Red peony | Horse pocket bell | Golden fruit | Huang Jing | Cang art | Dry ginger | Xiantao grass |
| 4 | Grass fruit | Gan Sui | Guati | Green wind rattan | Lugan | Dragon bile | Heaven fairy rattan | Epitarium | Ginkgo leaves | Even money grass | Know mother | Soap pod |
| 5 | Black tiger | Golden fruit | Opium courage | Mab | Man jing zi | Man jing zi | Mutong | Buddha hand | Jade bamboo | Wild chrysanthemum | Plate blue root | Flat bean flower |
| 6 | A thousand years of health | Perrin | White head weng | Chinese wolfberry | Prince ginseng | Pour buckle grass | Perrin | Fish fishy grass | Bench Solanum | Black tiger | White flower vegetables | Songziren |
| 7 | Ginger yellow | Stream grass | The dragon sunflower | Pepper | Xin Yi | Bone broken fill | Nansha ginseng | Sichuan Wu | Mu Xiang | Half branch lotus | Green wind rattan | Melo |
| 8 | Orange red | Credit wood | Corn must | Caenorhabditis elegans | Lily | Lingling fragrance | Dry ginger | White before | neem skin | Golden boiling grass | Big green leaves | White and |
| 9 | Sap Angle thorn | Epitarium | Ping Bei mother | Sand kernel | One hundred | The elder brother king root | Search for bone wind | Wild chrysanthemum | Prince ginseng | Grass fruit | shepherd's purse | Nuomi root |
| 10 | Even money grass | White head weng | Bitter ginseng | Know mother | Light heart grass | neem skin | Gardenia | Tianshan snow lotus | Gan Song | Ginger yellow | Quinb | Atilon hemp |
| 11 | Purple flower didine | Lead the cow | Caenorhabditis elegans | Polygonum daqing leaf | Duck metatarsal | Ginkgo leaves | Sichuan cow knee | Credit wood | Xihe Liu | coriander flowers | Calcutrix | Bai Wei |
| 12 | Yin Chen | Mu Xiang | Hawthorn leaves | Angelica | Sichuan shot dry | The ripe yellow | Xin Yi | Gan Sui | Nansha ginseng | A yellow flower | The radish | Sky pollen |
| 13 | Ghost needle grass | Horse whip grass | Bull knee | Ginger | Bench Solanum | Floating ping | Big thistle | Rocky rattan | One hundred | Yin Chen | Cang ear son | The wooden turtle |
| 14 | Mujingzi | Citron | Secret flowers | Cang ear son | Sophora japonica | Lychee core | Huashan ginseng | Man jing zi | Xin Yi | Three white grass | Mawei lotus | Mountain mushroom |
| 15 | Acute subsp | Chicken vine | The green seal | Chuanxiong | Josephine | Xin Yi | Sand kernel | Citron | The halberd day | Side cypress leaves | Loquat leaves | Lotus room |
| 16 | The swelling and the wind | Fish fishy grass | Sanzhi | Before hu | Chinese rose | Qian grass | Chen PI | Bench Solanum | Qian grass | Gan Song | Ma Huang | Lotus must |
| 17 | The neem | Red qi | Mountain bean root | Onions white | Dragon bile | Jing mustard | White before | Caenorhabditis elegans | Wood butterfly | Citron | Mab | Tempe bean |
| 18 | Tujing skin | Quixote | Angle lotus | Big green leaves | Mu Xiang | Opium courage | Uyao | Uyao | White MAO root | Sap Angle thorn | mung bean | Three white grass |
| 19 | Gan Song | Big abdominal skin | Angle lotus | Rose | The elder brother king root | Caenorhabditis elegans | Up hemp | Bitter almond | Golden lantern | Tiger ear grass | Shingapi | Tiger ear grass |
| 20 | North sand ginseng | One hundred | Terror | Red flowers | Lychee core | Josephine | Mu Xiang | Bone broken fill | Wild chrysanthemum | Fairy crane grass | Xuan ginseng | Committee mausoleum dishes |

**Table S3. Results of collaborative filtration of drugs in Qingfei Paidu decoction.**

| Index | Pig ling | Guangxi branch | Bitter almond | Fu Ling | Ma Huang | White art | Guanghuoxiang | Chai Hu | Huang Qin | Winter flowers | The aster | Ginger | Half summer | Ze diarrhea | Yam | licorice | Chen PI | Fine Xin | Orange solid | gypsum |
| --- | --- | --- | --- | --- | --- | --- | --- | --- | --- | --- | --- | --- | --- | --- | --- | --- | --- | --- | --- | --- |
| 1 | Pink Bi Yan Zhang | Pepper | White before | Cat claw grass | Purple Su Zi | Silksand | Su Hexiang | Ping Bei mother | Huang Lian | Ping Bei mother | Changshan | Soil tuckahoe | Astragalus | Xuan ginseng | Acid jujube kernel | Perrin | Cang art | Pepper | Changshan | Five times the son |
| 2 | All kinds of flowers | Ganoderma lucidum | Tianshan snow lotus | White seal | shepherd's purse | Chicken bone grass | Iye | Corn must | North five flavor son | Tubei mother | Ocean golden flowers | Dry ginger | Tan Xiang | Soil tuckahoe | Black sesame seeds | coriander | Sea Jinsha | Ganoderma lucidum | The mountains are red | The corrugated |
| 3 | In front of the car grass | Lily | Ibel mother | The venison | The dogwood | Tubei mother | Horse pocket bell | Tubei mother | Acid jujube kernel | Tiannan star | Mountain bean root | Xuan ginseng | Tiannan star | Dry ginger | Mulberry leaves | Huang Jing | Iron line through bone grass | Lotus leaf | Uyao | Xiantao grass |
| 4 | No medicine | Chinese rose | Gan Sui | Ganoderma lucidum | Green wind rattan | Tiannan star | Heaven fairy rattan | The dragon sunflower | Credit wood | Chai Hu | Winter flowers | Stemon white | Silksand | Cattle yellow | Rohan fruit | Ginkgo leaves | Iron line through bone grass | Changshan | Guati | Soap pod |
| 5 | The cow is strong | The mountains are red | Golden fruit | Half the lotus | Mab | Ping Bei mother | Mutong | Lotus leaf | Bai Zhi | Chuan Bei mother | Caenorhabditis elegans | Cang ear son | Pepper | Ginger | Half summer | Jade bamboo | Big thistle | Guangxi branch | Lactobacillus | Flat bean flower |
| 6 | Chuan Mu Tong | Chicken vine | Perrin | Tribulus | Chinese wolfberry | Tan Xiang | Perrin | Chicken bone grass | Fish fishy grass | Piper seal | Silver wood Hu | Sand kernel | Prevent yourself | Cang ear son | Sand spines | Bench Solanum | Gan Song | Wind | Three seven | Songziren |
| 7 | White hair summer dry grass | Uyao | Stream grass | Tory tea | Pepper | Tribulus | Nansha ginseng | Secret flowers | Mulberry leaves | Red flowers | Three seven | The radish | Mulberry leaves | Western ginseng | Astragalus | Mu Xiang | North sand ginseng | Ocean golden flowers | Decay sauce grass | Melo |
| 8 | Wild chrysanthemum | North five flavor son | Credit wood | Yunzhi | Caenorhabditis elegans | Chun skin | Dry ginger | Winter flowers | Bitter ginseng | Ganoderma lucidum | The dragon sunflower | Winter flowers | Red flowers | Know mother | Ganoderma lucidum | neem skin | Perrin | Winter flowers | Gao Liang Jiang | White and |
| 9 | Bamboo Festival ginseng | Wind | Epitarium | Chicken bone grass | Sand kernel | Mulberry leaves | Search for bone wind | Mulberry leaves | Chinese rose | Mulberry leaves | The ripe yellow | Stone calamus | Mab | Stone calamus | Ginseng | Prince ginseng | Bench Solanum | Gao Liang Jiang | Chicken vine | Nuomi root |
| 10 | Huang Jing | One hundred | White head weng | Jin Yingzi | Know mother | YanHuSuo | Gardenia | The green seal | Big thistle | The dragon sunflower | Ping Bei mother | The dogwood | Yam | Sand kernel | Western ginseng | Gan Song | Xin Yi | Through the crowd | Fish fishy grass | Atilon hemp |
| 11 | Tujing skin | Ping Bei mother | Lead the cow | Duck metatarsal | Polygonum daqing leaf | Guan Huang Bai | Sichuan cow knee | Elshole | Guangxi branch | Half the lotus | Lei Gong rattan | Before hu | Piper seal | shepherd's purse | Jujube | Xihe Liu | The halberd day | Sea breeze rattan | Mountain bean root | Bai Wei |
| 12 | The lamp is fine | Epitarium | Mu Xiang | Citrate leaves | Angelica | Astragalus | Xin Yi | White seal | Dragon bile | Elshole | Lactobacillus | Half summer | Jujube | The dogwood | Red flowers | Nansha ginseng | A yellow flower | Chicken vine | Xu Changqing | Sky pollen |
| 13 | Snake bed | Appendix | Horse whip grass | Appendix | Ginger | Tory tea | Big thistle | Prevent yourself | Even warped | Ocean golden flowers | White art | Ze diarrhea | Sand spines | Water fly thistle | Cicc | One hundred | Yin Chen | Bitter ginseng | Perrin | The wooden turtle |
| 14 | Golden chrysanthemum | Decay sauce grass | Citron | Wang did not leave the line | Cang ear son | Appendix | Huashan ginseng | Jersey paint | The mountains are red | The aster | Wind | Yam | Burdock | Calcutrix | Malts | Xin Yi | Turn orange red | Fish fishy grass | Pepper | Mountain mushroom |
| 15 | Winter | Half the lotus | Chicken vine | Tubei mother | Chuanxiong | Secret flowers | Sand kernel | Chun skin | Lead the cow | A daughter | Rohan fruit | Know mother | Winter flowers | Yam | Soil tuckahoe | The halberd day | Lychee core | Caenorhabditis elegans | Pepper | Lotus room |
| 16 | Shi Wei | Elshole | Fish fishy grass | Chicory | Before hu | The green seal | Chen PI | Citrate leaves | Three seven | Prevent yourself | Wear heart lotus | Big green leaves | Ping Bei mother | Guan Huang Bai | Black beans | Qian grass | neem skin | Golden lotus | Caenorhabditis elegans | Lotus must |
| 17 | aloe vera | Winter flowers | Red qi | Corn must | Onions white | Silver wood Hu | White before | Appendix | Decay sauce grass | Appendix | Tiannan star | Quinb | Horse pocket bell | Quinb | Seaweed | Wood butterfly | Rozi | Huang Lian | White head weng | Tempe bean |
| 18 | The smelly sycamore | Man jing zi | Quixote | White fresh skin | Big green leaves | Burdock | Uyao | Bull knee | The dragon sunflower | Chicken bone grass | Chicken vine | Rose | YanHuSuo | Polygonum daqing leaf | Yunzhi | White MAO root | Up hemp | Uyao | Huang Lian | Three white grass |
| 19 | Beijing halberd | A daughter | Big abdominal skin | Gergan | Rose | The dragon sunflower | Up hemp | Decay sauce grass | Western ginseng | Wind | Jersey paint | Red flowers | Wu dogwood | Caenorhabditis elegans | Wang did not leave the line | Golden lantern | Black tiger | North five flavor son | Guangxi branch | Tiger ear grass |
| 20 | coriander flowers | Fish fishy grass | One hundred | The hemp yellow root | Red flowers | Wind | Mu Xiang | Tiannan star | Wear heart lotus | Rohan fruit | Golden lotus | Polygonum daqing leaf | Jersey paint | Chicken excrement rattan | Mab | Wild chrysanthemum | Tujing skin | Mountain bean root | Star anise | Committee mausoleum dishes |

**Table S4. Results of collaborative filtration of drugs in Jinhua Qinggan Granule.**

| Index | Bitter almond | Even warped | Ma Huang | Burdock | Artemia | Honeysuckle | Huang Qin | Mint | Know mother | Zhejiang Bei mother | licorice |
| --- | --- | --- | --- | --- | --- | --- | --- | --- | --- | --- | --- |
| 1 | White before | North five flavor son | Purple Su Zi | Tan Xiang | Summer withered grass | Caenorhabditis elegans | Huang Lian | Wild chrysanthemum | Polygonum daqing leaf | The green seal | Perrin |
| 2 | Tianshan snow lotus | Credit wood | shepherd's purse | Before hu | Sichuan shot dry | Party participation | North five flavor son | Half branch lotus | Big green leaves | Tribulus | coriander |
| 3 | Ibel mother | Fish fishy grass | The dogwood | Mulberry leaves | Red peony | Golden lotus | Acid jujube kernel | Rob hemp leaf | shepherd's purse | Mulberry leaves | Huang Jing |
| 4 | Gan Sui | Huang Lian | Green wind rattan | Pepper | Dragon bile | Changshan | Credit wood | A yellow flower | Green wind rattan | Tubei mother | Ginkgo leaves |
| 5 | Golden fruit | Mountain bean root | Mab | Chuan Bei mother | Man jing zi | Before hu | Bai Zhi | Black tiger | Plate blue root | Astragalus | Jade bamboo |
| 6 | Perrin | White before | Chinese wolfberry | Ping Bei mother | Pour buckle grass | Even warped | Fish fishy grass | Sap Angle thorn | Cang ear son | The dragon sunflower | Bench Solanum |
| 7 | Stream grass | Changshan | Pepper | Amarlane | Bone broken fill | Decay sauce grass | Mulberry leaves | Even money grass | Caenorhabditis elegans | Through the crowd | Mu Xiang |
| 8 | Credit wood | Bai Zhi | Caenorhabditis elegans | The green seal | Lingling fragrance | Rohan fruit | Bitter ginseng | Purple flower didine | Party participation | YanHuSuo | neem skin |
| 9 | Epitarium | Honeysuckle | Sand kernel | Tubei mother | The elder brother king root | Orange stalk | Chinese rose | Mujingzi | White fruit | Guan Huang Bai | Prince ginseng |
| 10 | White head weng | Horse whip grass | Know mother | White art | neem skin | Winter flowers | Big thistle | The neem | Ma Huang | Prevent yourself | Gan Song |
| 11 | Lead the cow | Huang Yaozi | Polygonum daqing leaf | Orange stalk | Ginkgo leaves | Purple Sue | Guangxi branch | Tujing skin | Xuan ginseng | Golden lotus | Xihe Liu |
| 12 | Mu Xiang | Ibel mother | Angelica | Winter flowers | The ripe yellow | Ink drought lotus | Dragon bile | North sand ginseng | Honeysuckle | Cat claw grass | Nansha ginseng |
| 13 | Horse whip grass | Gan Sui | Ginger | Stemon white | Floating ping | Chuan Bei mother | Even warped | Xihe Liu | Ginger | Wang did not leave the line | One hundred |
| 14 | Citron | Chicken vine | Cang ear son | Tribulus | Lychee core | Know mother | The mountains are red | Snake bed | Sand kernel | Secret flowers | Xin Yi |
| 15 | Chicken vine | Orange solid | Chuanxiong | Black beans | Xin Yi | Huang Lian | Lead the cow | Yu Jin | Stemon white | Make the gentleman | The halberd day |
| 16 | Fish fishy grass | Golden lotus | Before hu | Seaweed | Qian grass | Mawei lotus | Three seven | The tortotortoisis | The mustard | Calcutrix | Qian grass |
| 17 | Red qi | White head weng | Onions white | Red flowers | Jing mustard | Madonna | Decay sauce grass | Citron | Li Zi | Duck metatarsal | Wood butterfly |
| 18 | Quixote | Guangxi branch | Big green leaves | Ginseng | Opium courage | Three seven | The dragon sunflower | Cang art | Golden lotus | Ping Bei mother | White MAO root |
| 19 | Big abdominal skin | Lovesickness | Rose | Half summer | Caenorhabditis elegans | Burdock | Western ginseng | Fairy crane grass | The dogwood | Tianzhu yellow | Golden lantern |
| 20 | One hundred | Perrin | Red flowers | Silksand | Josephine | Angelica | Wear heart lotus | Side cypress leaves | Cattle yellow | Bitter ginseng | Wild chrysanthemum |

**Table S5. Results of collaborative filtration of drugs in Huashi Baidu recipe.**

| Index | rhizoma atractylodis | semen armeniacae amarae | Poria cocos | Mangnolia officinalis | Chinese ephedra | rheum officinale | Pogostemon cablin | Pinellia ternata | Amomum tsao-ko | liquorice | semen lepidii | Astragalus mongholicus | radix paeoniae rubrathe root of common peony | gyp |
| --- | --- | --- | --- | --- | --- | --- | --- | --- | --- | --- | --- | --- | --- | --- |
| 1 | fructus cnidii | Cynanchum glaucescens | ternate buttercup root | red ginseng | perillae,fructus | azedarach | storax | Astragalus mongholicus | kaki calyx | eupatorium | mustard | Pinellia ternata | fructus viticis | Chinese gall |
| 2 | solidago decurrens | Tianshan snow lotus | radix ampelopsis | cattail pollen | shepherd's purse | radices saussureae | folium artemisiae argyi | sanders | rhizoma atractylodis | coriander | Folium Polygoni Tinctorii | Canton love-pea vine | selfheal | concha arcae |
| 3 | mother chrysanthemum | Fritillaria pallidiflora Schrek. | Citrus trifoliata | Hypericum japonicum Thumb. | fructus corni | rhizoma sparganii | Fructus Aristolochiae | Arisaema consanguineum Schott | potentilla chinensis | solomon's seal | rhizoma zingiberis | folium mori | madder | purslane speedwellHerba |
| 4 | Amomum tsao-ko | Euphorbia kansui | glossy ganoderma | cat's-foot | caulis sinomenii | Java brucea | herba aristolochiae | silkworm excrement | rhizoma nardostachyos | ginkgo leaf | rhizoma anemarrhenae | Arisaema consanguineum Schott | drynaria rhizome | semen gleditsiae sinensis |
| 5 | Black tiger | Golden fruit | Chinese lobelia | radices echinopsis latifolii | bovista | lignum et folium trachelospermi | Akebia quinata Decne. | cayenne pepper | fennel | radix polygonati officinalis | isatis root | glossy ganoderma | Artemisia apiacea | lablab,flos |
| 6 | rhizoma homalonemae | eupatorium | Fructus Tribuli | rhizoma atractylodis | the fruit of Chinese wolfberry | drynaria rhizome | eupatorium | the root of fangji | Golden chrysanthemum | piper cubeba | semen gynandropsis | bighead atractylodes rhizome | semen litchi | SEMEN PINI KORAIENSIS |
| 7 | rhizoma curcumae longae | lycopodium clavatum | broadleaf holly leaf | Black tiger | cayenne pepper | the bark of ash | adenophora tetraphylla | folium mori | negundo chastetree fruit | radices saussureae | caulis sinomenii | silkworm excrement | radix gentianae | semen melo |
| 8 | tangerine | leatherleaf mahonia | rainbow conk | chrysanthemum | scandent hop | cymose buckwheat rhizome | rhizoma zingiberis | Carthamus tinctorious | citronella | azedarach | herba taching | Fritillaria thunbergii Miq. | lignum millettiae | Bletilla hyacinthina Reichb. |
| 9 | spina gleditsiae | Aceranthus sagittatus S. et Z. | Canton love-pea vine | fructus cnidii | fructus amomi | herba cepbalanoplosis segeti | berba aristolochiae mollissimae | bovista | pericarpium citri reticulatae viride | radix pseudostellariae | shepherd's purse | Fructus Tribuli | flos magnoliae liliflorae | orizae,radix |
| 10 | Nepeta glechoma Benth. | the root of Chinese pulsatilla | fructus rosae laevigatae | Areca catechu | rhizoma anemarrhenae | airpotato yam | Cape jasmine | Chinese yam | tangerine leaf | rhizoma nardostachyos | sea-tangle | the root of fangji | cymose buckwheat rhizome | chingma abutilon seed |
| 11 | tokyo violet herb | kaladana | Commelina communis L. | tangerine | Folium Polygoni Tinctorii | exocarpium citri grandis | Cyathnla capitata Moq. | long pepper | potentilla chinensis | Chinese tamarisk tops | Trichosanthes kirilowii Maxim | Semen sesami nigrum | lily | Cynanchum atratum Bge. |
| 12 | oriental wormwood | radices saussureae | holly leaf | periplocae,cortex | Angelica sinensis | Golden fruit | flos magnoliae liliflorae | Chinese-date | Black tiger | adenophora tetraphylla | raphani,semen | rhizoma bolbostemmae | saffron | radices trichosanthis |
| 13 | sticktight | herba verbenae | radix aconiti carmichaeli | solidago decurrens | ginger | piper cubeba | Circium japonicum | sea-buckthorn | fructus cnidii | the tuber of stemona | cocklebur fruit | Chinese yam | Japanese ardisia | Momordica cochinchinensis |
| 14 | negundo chastetree fruit | fructus citri | the seed of cowherb | folium panacis japonici cum caule | cocklebur fruit | Flos Sophorae | Physochlaina macrophylla | fructus arctii | RADIX SMILACIS SIEBOLDI | flos magnoliae liliflorae | thalictrum root | fritillary bulb | ginkgo leaf | pseudobulbus cremastrae seu pleiones |
| 15 | of impatient disposition | lignum millettiae | rhizoma bolbostemmae | rhizoma nardostachyos | Ligusticum wallichii | radix gentianae | fructus amomi | flos farfarae | Hydnocarpus anthelmintica | Morinda officinalis | folia eriobotryae | semen zizyphi spinosae | myrrh | receptaculum nelumbinis |
| 16 | zhongjiefeng | cordate houttuynia | witloof | rhizoma zingiberis | Angelica decursiva | pokeberry root | dried tangerine or orange peel | fritillary bulb | ramuli euonymi | madder | Chinese ephedra | Chinese-date | fructus citri | Stamen Nelumbinis |
| 17 | fructus meliae | hedysari,radix | stigmata maydis | myrrh | very light blue | fructus dauci carotae | Cynanchum glaucescens | Fructus Aristolochiae | tangerine pith | oroxylum indicum | bovista | rainbow conk | radices paeoniae alba | semen sojae praeparatum |
| 18 | golden larch bark | chebule | cortex dictam | oil orange | herba taching | fructus viticis | the root of three-nerved spicebush | corydalis tuber | buzhaye | rhizoma imperatae | urad | radix aconiti carmichaeli | Coptis chinensis | saururus chinensis |
| 19 | rhizoma nardostachyos | the shell of areca nut | the root of kudzu vine | mother chrysanthemum | rose | Indian stringbush root | rattletop | evodia rutaecarpa | Nepeta glechoma Benth. | wintercherry fruit or calyx | periplocae,cortex | butterflybush flower | cassia twig | Saxifraga stolonifera |
| 20 | radix glehniae | the tuber of stemona | radices ephedrae | Acorus gramineus Soland. | Carthamus tinctorious | leatherleaf mahonia | radices saussureae | wartwort | Saxifraga stolonifera | mother chrysanthemum | radix scrophulariae | Chinese lobelia | common achyranthes herb | potentilla chinensis |
